# Pre-hospital and intrahospital workflow optimization for patients with suspected ischemic stroke due to large vessel occlusion - findings from a tertiary care facility

**DOI:** 10.1186/s12883-022-03033-1

**Published:** 2022-12-22

**Authors:** Stefan Krebs, Julia Ferrari, Alice Schürer, Astrid Chiari, Christian Neumann, Wilfried Lang, Marek Sykora

**Affiliations:** 1 Department of Neurology, St. John’s Hospital, Johannes Von Gott Platz 1, 1020 Vienna, Austria; 2 Medical Faculty, Sigmund Freud University, Vienna, Austria; 3 Department of Anesthesiology, St. John’s Hospital, Vienna, Austria; 4 Department of Radiology, St. John’s Hospital, Vienna, Austria

**Keywords:** LVO, Mechanical thrombectomy, Ischaemic stroke, Workflow

## Abstract

**Background:**

The efficacy of recanalization treatment in patients with ischemic stroke due to large vessel occlusion (LVO) is highly time dependent. We aimed to investigate the effects of an optimization of prehospital and intrahospital pathways on time metrics and efficacy of endovascular treatment in ischemic stroke due to LVO.

**Methods:**

Patients treated with mechanical thrombectomy (MT) at the Hospital of St. John of God Vienna, Austria, between 2013 and 2020 were extracted from the Austrian Stroke Unit Registry. Study endpoints including time metrics, early neurological improvement and functional outcome measured by modified Rankin Scale (mRS) at 3 months were compared before and after optimization of prehospital and intrahospital pathways.

**Results:**

Two hundred ninety-nine patients were treated with MT during the study period, 94 before and 205 after the workflow optimization. Workflow optimization was significantly associated with time metrics improvement (door to groin puncture time 45 versus 31 min; *p* < 0.001), rates of neurological improvement (NIHSS ≥ 8: 30 (35%) vs. 70 (47%), *p* = 0.04) and radiological outcome (TICI ≥ 2b: 71 (75%) versus 153 (87%); *p* = 0.013). Functional outcome (mRS 0–2: 17 (18%) versus 57 (28%); *p* = 0.067) and mortality (34 (37%) versus 54 (32%); *p* = 0.450) at 3 months showed a non-significant trend in the later time period group.

**Conclusion:**

The implementation of workflow optimization was associated a significant reduction of intrahospital time delays and improvement of neurological and radiological outcomes.

## Background

Mechanical thrombectomy (MT) is an effective and proven treatment in patient’s ischemic stroke due to large vessel occlusion (LVO) [1]. The benefit of the recanalization is highly time dependent. Various concepts exist to reduce prehospital time metrics [2]. Additionally, of importance is to optimize intrahospital workflows within a multidisciplinary approach. Current guidelines recommend as targets a door to needle time (DNT) of 30–60 min and a door to reperfusion time (DRT) between 90 and 120 min [3]. Study designs that investigate workflow optimization were very heterogeneous. A meta-analysis of prehospital optimization reported an increase of IV tPA treatment (IVT) rate, shorter time metrics for LVO patients [4]. However, mortality and rate of good functional outcome did not differ significantly [4]. Intrahospital workflow optimization was associated significantly with shorter door to needle times, lower mortality, lower rates of symptomatic intracranial hemorrhage and higher reperfusion rates within 60 min [5]. In 2016, we optimized our prehospital and intrahospital workflows to achieve a time to treatment reduction using as standard operating procedure. The aim of our study was to investigate the impact of this optimization on treatment times metrics, radiological and functional outcome.

## Methods

The study population consisted of consecutive acute ischemic stroke patients treated between 2013 and 2020 in a comprehensive stroke center at the Hospital of St. John of God in Vienna, Austria. Patient’s data were extracted from the Austrian Stroke Unit Registry (ASUR). ASUR is a nationwide prospective registry of the Austrian stroke unit network founded by the Federal Ministry of Health. Anonymized data on baseline characteristics, risk factors and etiology, acute management, and functional outcome at discharge and at 3 months are registered for all patients admitted to one of currently 39 stroke units in Austria. Data collection and clinical ratings are performed by experienced stroke neurologists using standardized definitions of variables and scores. To ensure high data quality, immediate electronic data entry is performed. The web-based database includes online plausibility checks and a help function. Biannual educational meetings serve to guarantee uniform data documentation. A detailed methodological description has been published previously [6].

For the means of current study, we included only patients directly referred to our stroke center. Following variables were extracted from the registry and entered the analysis: age, sex, IV tPA treatment (IVT) rate, spontaneous intracranial hemorrhage (SICH) occurrence according to ECASS 3 [7] criteria, National Institute of Health Stroke Scale (NIHSS) at admission and discharge from stroke unit, modified Rankin Scale (mRS) – pre-stroke, at discharge and at 3 months follow-up, risk factors (hypertension, diabetes, atrial fibrillation), onset to door time (ODT), onset to recanalization time (ORT), door to groin puncture time (DPT) and door to recanalization time (DRT). We compared patients treated with MT before and after optimization of the prehospital and intrahospital workflows (2013–2016 versus 2016–2020). In 2016, an interdisciplinary standard operating procedure (SOP) has been designed and activated encompassing following points:

1. Prehospital notification using a LVO identification score (Austrian Prehospital stroke Scale (APSS, [8]) by the emergency medical service (EMS) car
2. Prehospital electronic collection of patient’s data including medical history and actual medication using a direct view-only access to the central EMS system.
3. Electronic pre-administration of patient’s admission including pre-administration of neuroimaging, endovascular and anesthetic procedures
4. Reducing of blood sampling at admission
5. Definition of specific roles and tasks for all team members using a dedicated SOP
6. Definition of specific anesthesiologist and interventional radiologist on call for the MT procedure.
7. Preliminary alarm for all team members on hospital admission in case of patients with severe stroke symptoms (APSS ≥ 4) including alerting of the angio suite program manager
8. Definite alarm for all team member at the time of identification of LVO in neuroimaging with the code urgency equivalent to cardiopulmonary arrest
9. IVT in the computertomography (CT) room, equipment for monitoring and IVT stationary in CT room
10. Additional anesthesiologist (resident) for the angio suite
11. Rapid sequence intubation standards and rigid blood pressure targets defined by dedicated standard operating procedure
12. Process monitoring and feedback rounds

For the evaluation of effects of optimization following study endpoints have been defined:

- Procedure endpoints: Changes of times including ODT, DPT, DRT
- Early neurological improvement between admission NIHSS and NIHSS at 24–48 h more or equal 8 points
- Functional outcome (dichotomized mRS 0–2) and mortality at 3 month follow up
- Radiological outcome (Thrombolysis in cerebral infarction (TICI) 2b-3) after MT

Data of patients treated with MT in other comprehensive stroke centers in Austria were extracted from ASUR and used as control group in a sensitivity analysis.

### Statistics

Results are presented as median, range, and interquartile range (IQR) for continuous variables, while categorical variables are summarized by absolute x (n) and relative (%) frequencies*. *Patients were categorized one group before and after implementation of new workflows. Mann–Whitney U-test was used to compare the locations of continuous and ordinal variables without a normal distribution. Pearson's Chi Square test was comparing frequency and distribution of categorical variables. All statistics were performed using statistical software SPSS, version 27, IBM.

### Ethics

The Austrian stroke unit registry is part of a governmental quality assessment program for nationwide stroke care and is financed by the Federal Ministry of Health. Prospective data entry is mandatory for stroke units. All data are anonymized and centrally administered by the Gesundheit Oesterreich GmbH—the national research and planning institute for health care, a competence and funding center of health promotion. All scientific analyses included in this study were approved and supervised by a national academic review board. Based on this setting, informed consent for patients enrolled in the registry was waived.

## Results

### Study population

Two hundred ninety-nine patients undergoing MT in our stroke center entered the analysis. In this cohort 56% were female with a mean age of 73 (Standard deviation (SD 14) and a median admission NIHSS of 16 (IQR 11, 20). We compared 94 patients treated before and 205 treated after implementation of the workflow optimization protocol. Baseline characteristics differed significantly between both groups. Patients in the after group showed higher rates of risk factors (hypertension 77% vs. 82%, *p* = 0.070; smoking 15% vs. 24%; *p* = 0.010; myocardial infarction 11% vs. 19%, p = 0.001). Patients in this group had significant less severe strokes (admission NIHSS 18 vs 16, *p* = 0.001) and equal levels of pre-stroke independence (mRS 0 74% vs. 69% 0, *p* = 0.302). The rate of IV tPA administration was significant higher in the before group (76% vs. 54%, *p* = 0.001). The locations of the artery occlusions were equally distributed in both groups. (Table 1).

**Table 1:** Comparison of baseline patient characteristics before and after optimization of the prehospital and intrahospital workflows

	Before group (2013-2016) *N* = 94	After group (2016-2020) *N* = 205	*P*-value
**Baseline characteristics**			
Sex (female)	60 (64%)	108 (53%)	0.071
Age	76 (66, 83)	75 (64, 82)	0.403
NIHSS on admission	18 (14, 22)	16 (9, 20)	0.001
Previous stroke	10 (11%)	39 (20%)	0.051
Wake up stroke	12 (13%)	34 (17%)	0.395
pmRS	0 (0, 0)	0 (0, 1)	0.302
Thrombolysis prior to MT	72 (76%)	111 (54%)	0.001
**Risk factors**			
Hypertension	72 (77%)	168 (82%)	0.070
Diabetes	20 (21%)	38 (19%)	0.280
Hyperlipidemia	54 (57%)	120 (58%)	0.154
Myocardial infarction	10 (11%)	39 (19%)	0.001
Smoking	14 (15%)	50 (24%)	0.011
**Locations**			
Proximal ACI	5 (5%)	9 (4%)	0.317
M1	53 (56%)	123 (60%)	
M2	15 (16%)	43 (21%)	
Basilar	1 (1%)	3 (15%)	
Distal ACI	12 (16%)	27 (13%)	

*NIHSS* National Institute of Health Stroke Scale, *pmRS* Premorbid Rankin Score

### Time metrics and outcomes

The implementation of the new workflow led to a significant decrease of all intrahospital pathways (DPT 45 min vs. 31 min (*p* < 0.001) and DRT 102 versus 75 min, *p* < 0.001).

The rates of wake-up strokes were equal in both groups (12 (13%) vs. 34 (17%); *p* = 0.395). Patients in the after group showed significant better radiological outcome (TICI ≥ 2b 71 (75%) versus 153 (87%); *p* = 0.013) and early neurological improvement of NIHSS ≥ 8 (30(35%) vs. 70(47%), *p* = 0.04). SICH occurred equally in both groups (n(%): 5(5%) vs. 9(4%), *p* = 0.724). Functional outcome (mRS 0–2: 17 (18%) vs. 57 (28%); *p* = 0.067) and mortality (34 (37%) vs. 54 (32%); *p* = 0.450) at 3 months showed a positive trend, however, did not differ significantly. (Table 2).

**Table 2:** Comparison of outcome parameters before and after optimization of the prehospital and intrahospital workflows

Time-frames (in minutes)	Before group (2013-2016)	After group (2016-2020)	*P*-Values
Onset to door time	60 (45, 75)	66 (53, 104)	0.170
Door to puncture time	45 (37, 52)	31 (25, 38)	< 0.001
Door to recanalization time	102 (85, 126)	75 (51, 100)	< 0.001
Functional and safety outcomes	Before group	After group	
NIHSS improvement ≥ 4	37 (42%)	90 (61%)	0.005
NIHSS improvement ≥ 8	31 (35%)	70 (47%)	0.04
mRS 0–2 at 3 months FU	17 (18%)	57 (28%)	0.067
SICH	5 (5%)	9 (4%)	0.724
TICI ≥ 2b	71 (75%)	153 (87%)	0.013
Mortality at 3 months FU	34 (37%)	54 (32%)	0.450

*NIHSS* National Institute of Health Stroke Scale, *mRS* Modified Rankin Score, *SICH* Spontaneous intracranial hemorrhage

#### Sensitivity analysis using control group including data of patients treated using thrombectomy in other comprehensive stroke centers in Austria

Three thousand eight hundred twenty-four patients treated with thrombectomy in other comprehensive stroke centers between 2013 and 2020 in Austria were used as a control group. The control group included 1898 (49%) female with a mean age of 74 (SD 14). These patients showed comparable onset to door times, however, significant longer intrahospital times as compared to patients treated at Hospital St. John of God (intervention group) (ODT 63 min vs. 63 min, *p* = 0.166; DPT 99 min vs. 35 min, *p* < 0.001; DRT 146 min vs. 87 min, *p* < 0.001). To verify the impact of implementation of the new workflow in our center, we compared with workflow metrics before and after 2016 cumulatively in other stroke centers: The population did not differ significantly in age (n: 73(SD 14) vs 72 (SD 14), *p* = 0.079), sex (n(%): 168(56%) vs. 1898 (50%); *p* = 0.088) and baseline characteristics. In contrast to the findings of our center, the time metrics of intrahospital pathways before and after 2016 (DPT 104 versus 98 min, *p* = 0.190; DRT 151 versus 141 min, *p* = 0.100) decreased, however, the difference was not significant.

## Discussion

We demonstrated that the optimization of intrahospital workflows may lead to a significant reduction of intrahospital time metrics. These results were stable between 2016 and 2020 and were not affected significantly by the COVID19 pandemic. In contrat, intrahospital workflow time benchmarks changed only non-significantly in other centers.

Furthermore, our data showed significant higher rates of neurological improvement and radiological outcome in the after group. Safety endpoint (SICH occurrence) did not differ significantly and was in line with large registry observations [9]. These effects translated into a non-significant trend to better functional outcome at 3 months.

The rate of bridging thrombolysis decreased over time significantly. After the benefits of MT has been proven by MR CLEAN et al. [1] and other trials [10–13] the rate of MT was increasing and more patients with contraindication to IVT were selected for MT. During the study period the role of IVT in front of MT was controversial [14], we do not suppose it would have affected our results in means of functional outcome.

Every part of the stroke pathways was optimized. We implemented a score (APSS [8]) to detect patients with suspected LVO in the prehospital setting. The reduction of pre-hospital time metrics using APSS has been published previously [15].

The highest potential for pathway optimization between imaging and groin puncture was documented in previous studies [5]. The immediate alert of the interventional radiologist and the anesthesiology team at the time point of the LVO detection seem to reduce the potential time delay in this time window.

Current guidelines recommended a door to puncture time under 90 min [2]. Our center reached significant shorter door to puncture times already before optimization (45 min versus 100 min, *p* < 0.001) and also significant shorter door to puncture time as compared to the mean of other centers (mean DPT 35 min vs. 99 min, *p* < 0.001) (data not shown). After implementation of the optimized pathways in our center time metrics could be reduced further for another 14 min. Other European networks reported a reduction of DPT for 15 min after structured stroke simulation [16]. Furthermore, each 5 cases/year increase in MT case volume was associated with 3% shorter door to reperfusion time, up to a case volume of 40 per year [17].

All MTs were performed under general anesthesia using rapid sequence intubation and rigid blood pressure protocols. A meta-analysis of three small, randomized trials indicated more favorable functional outcome using general anesthesia [18] however, later meta-analyses suggested no outcome benefits over non-general anesthesia techniques [19] leaving this topic basically unresolved.

Several limitations have to be mentioned. The indication for MT and treatment time window has broadened significantly during the study period [20] and led to higher rates of advanced neuroimaging and higher MT rates. Together with the learning curve of interventionists and changing endovascular techniques and devices during the study period these factors comprise important unmeasured confounders. Further limitations arise from the retrospective, non-randomized character of the study. Thus, our results have to be interpreted with caution and regard to the above-mentioned limitations. On the other hand, the strength of our study is the rigorously collected prospective dataset reflecting closely the real-world setting in hospitalized acute stroke patients.

## Conclusion

Optimization of intrahospital workflows was associated to a significant reduction of time metrics and higher rates of neurological improvement.

## Data Availability

The datasets used and/or analyzed during the current study available from the corresponding author on reasonable request.
